# Clinical course and prognostic factors of *Pneumocystis* pneumonia with respiratory failure in non-HIV patients

**DOI:** 10.3389/fcimb.2024.1380494

**Published:** 2024-07-10

**Authors:** Jun Li, Xiangdong Mu, Haichao Li, Xinmin Liu

**Affiliations:** ^1^ Department of Respiratory and Critical Care Medicine, Beijing Tsinghua Changgung Hospital, School of Clinical Medicine, Tsinghua University, Beijing, China; ^2^ Department of Geriatrics, Peking University First Hospital, Beijing, China; ^3^ Department of Respiratory and Critical Care Medicine, Peking University First Hospital, Beijing, China

**Keywords:** *Pneumocystis* pneumonia, non-human immunodeficiency virus, respiratory failure, prognostic factor, risk factor

## Abstract

**Background:**

Compared with *Human Immunodeficiency Virus* (HIV) patients, non-HIV patients with *Pneumocystis* pneumonia (PCP) have more rapid onset, more rapid progression, and higher mortality.

**Objectives:**

To investigate the predictive value of variables obtained upon hospital admission for in-hospital death and 90-day outcomes in non-HIV-PCP patients with respiratory failure (RF).

**Methods:**

This was a single center retrospective study in a tertiary care institution over 15 years. It included all adults inpatients (≥18 years old) with laboratory confirmed non-HIV-PCP with RF who were discharged or died from Peking University First Hospital between April 1st, 2007 and November 1st, 2022. Epidemiological, clinical, laboratory, imaging and outcome data were collected from patient records.

**Results:**

In this study, a total of 146 non-HIV-PCP patients with RF were included. There were 57 patients (39%) died during hospitalization, 44 patients (53%) died in Intensive care unit (ICU). A total of 137 patients completed 90 days of follow-up, of which 58 (42.3%) died. The multivariable regression analysis revealed that a CD8^+^ T cell count <115/μl (*P*=0.009), bronchoalveolar lavage fluid (BALF)-neutrophil percentage ≥50% (*P*=0.047), the time from corticosteroids withdrawal to symptom onset ≤5 days (*P*=0.012), and the time from visit to initiation of sulfonamides ≥2 days (*P*=0.011) were independent risk factors for in-hospital death. Furthermore, a CD8^+^ T cell count < 115/μl (*P*=0.001) and the time from visit to initiation of sulfonamides therapy ≥2 days (*P*=0.033) was independently associated with 90-day all-cause death.

**Conclusions:**

A low CD8^+^ T cell count in peripheral blood, a high percentage of BALF-neutrophils, a short time from corticosteroids withdrawal to symptom onset, and a long time from visit to initiation of sulfonamides are associated with poor prognosis in non-HIV-PCP patients with RF.

## Introduction

*Pneumocystis* pneumonia (PCP) is a serious fungal infection that primarily affects immunocompromised individuals, such as those with *Human Immunodeficiency Virus* (HIV)/acquired immune deficiency syndrome (AIDS), cancer, or organ transplant recipients. With the extensive clinical application of tumor chemoradiotherapy, corticosteroids and immunosuppressants, the susceptible population of *Pneumocystis jirovecii* has gradually shifted from HIV infected individuals to non-HIV infected individuals (Buchacz et al., 2016; Pegorie et al., 2017; Xue et al., 2023). Respiratory failure (RF) in non-HIV related PCP patients is the most common cause of intensive care unit (ICU) admission and an independent risk factor for death (Maschmeyer et al., 2016), with a mortality rate of 50%-80% (Festic et al., 2005; Limper et al., 2017).

The clinical course of PCP varies between HIV and non-HIV infected patients. Generally, non-HIV-PCP patients exhibit more abrupt RF, while HIV infected patients have more insidious clinical manifestations (Monnet et al., 2008; Ko et al., 2014). This is related to the pathogen burden and inflammatory response in the lungs of HIV and non-HIV infected patients (Ziefer and Abramowitz, 1989). Studies have indicated that at least two-thirds of PCP patients require mechanical ventilation, which is associated with higher in-hospital mortality (Curtis et al., 2000; Mansharamani et al., 2000b; Festic et al., 2005; Miller et al., 2006). Consequently, non-HIV-infected patients with RF requiring mechanical ventilation have worse clinical outcomes than HIV-infected patients (Gaborit et al., 2019). Previous studies on prognostic risk factors in non-HIV-PCP patients with RF have identified high acute physiology and chronic health evaluation III (APACHE III) score, corticosteroid use before diagnosis of PCP, severity of disease, underlying disease, initial anti-PCP treatment failure, delayed intubation, elevated end-expiratory positive pressure on day 3, long duration of positive airway pressure ventilation, and pneumothorax were independent risk factors for poor prognosis (Festic et al., 2005; Boonsarngsuk et al., 2009; Ko et al., 2014; Burghi et al., 2021). These studies mainly focused on the influence of patient factors, laboratory indicators and treatment on the clinical outcome of patients (Kim et al., 2014). When patients develop adverse events such as RF, tracheal intubation, and ICU admission, the management of patients with PCP will be more difficult, and it is particularly important to pay attention to the occurrence of critical node events and to find risk factors affecting the prognosis. At present, there are few studies on the prognosis of non-HIV-PCP patients with RF, and the relationship between important event nodes in the clinical course and prognosis is not clear.

Therefore, we described and analyzed the clinical data of 146 non-HIV-PCP patients with RF in detail, aiming to find the risk factors affecting the prognosis of patients with RF through the clinical course of such patients and the changes in the time nodes of relevant important events, so as to optimize the clinical diagnosis and treatment, and provide the basis for improving the diagnosis and treatment of the disease and reducing the mortality.

## Methods

### Study design and population

We performed a retrospective analysis of adult patients with non-HIV-PCP who were admitted to Peking University First Hospital requiring treatment for RF from April 2007 to October 2022. Patients who met the following criteria were enrolled in the study (Xue et al., 2023): HIV test is negative (Pegorie et al., 2017); Diagnostic criteria for PCP (Enomoto et al., 2010; Ainoda et al., 2012; Vogel et al., 2012; Ricciardi et al., 2017): ① Cough, fever, dyspnea or hypoxemia and other clinical manifestations; ② Imaging findings consistent with PCP, such as ground-glass changes in both lungs or diffuse interstitial infiltration; ③ *Pneumocystis jirovecii* was detected in bronchoalveolar lavage fluid (BALF), sputum, bronchial flushing fluid, bronchial secretions and lung tissue (Buchacz et al., 2016); The definition of RF (Roussos and Koutsoukou, 2003).

### Data collection

Clinical data on each patient’s demographic characteristics, past history, underlying disease, laboratory indicators, microbiology, radiology, treatment and prognosis were collected by consulting electronic medical records. The date of diagnosis was defined as the date of microbial confirmation. The time from presentation to initiation of sulfonamides therapy referred to the time interval between outpatient, emergency treatment or direct admission to trimethoprim-sulfamethoxazole (TMP-SMX). A history of corticosteroids use referred to the use of corticosteroids within one month prior to admission for PCP. All available information related to dosage and duration need to be documented, and the dosage of different types of corticosteroids should be converted to equivalent doses of prednisone.

### Statistical analysis

In the study, measurement data conforming to normal distribution were expressed as mean ± standard deviation (x ± s), and t test was used to compare differences between groups. Measurement data of non-normal distribution were expressed as median (IQR), and comparison between two groups was performed by non-parametric Mann-Whitney U test. The rate of counting data (%) was expressed by chi-square test or Fisher exact test. The optimal cut-off value was determined by Receiver Operating Characteristic (ROC) curve, univariate and multivariate logistic regression was used to analyze the relationship between variables and poor prognosis. Kaplan-Meier survival curve was used to evaluate the 90-day survival of variables. Univariate and multivariate cox proportional hazard regression models were used to analyze the relationship between variables and 90-day poor prognosis. When *P* < 0.05, the difference was statistically significant. SPSS 23.0 and Graphpad Prism 8 were used for data statistics and analysis.

## Results

### Demographics of non-HIV-PCP patients with respiratory failure

**Figure 1:**
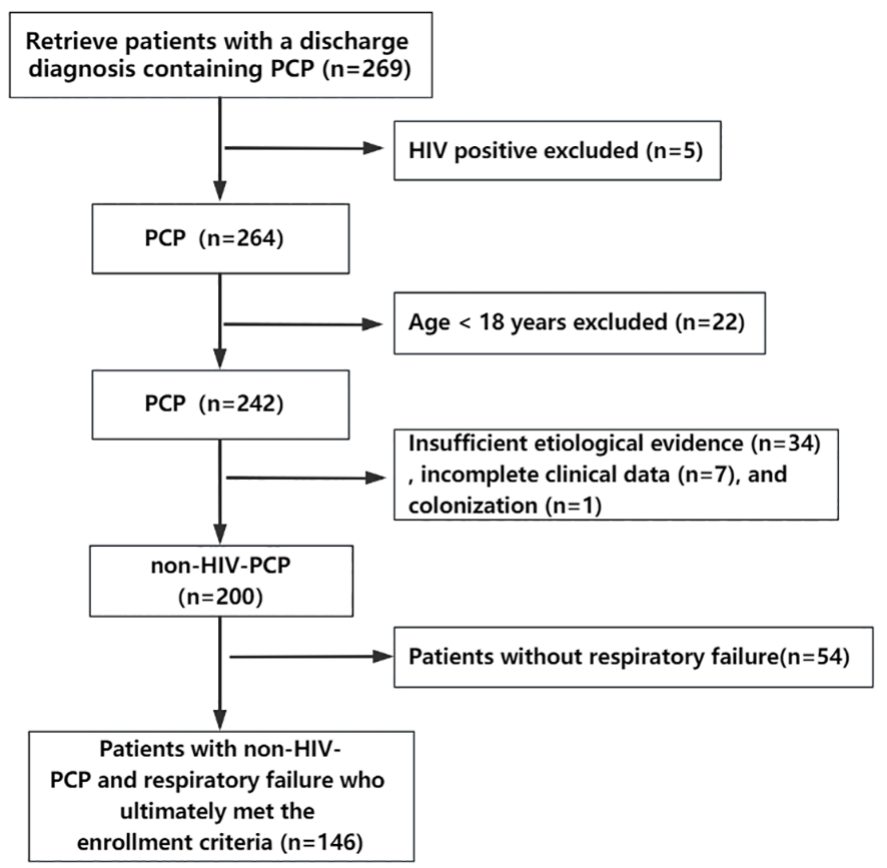
Flow chart of the study. A total of 269 patients diagnosed with PCP from April 2007 to October 2022 were retrieved through the electronic medical record system, excluding 5 HIV-positive patients, 22 patients under the age of 18, 34 patients with insufficient etiological evidence, 7 patients with incomplete clinical data, 1 patient with colonization, and 54 patients with non-respiratory failure. Finally, 146 patients meeting the criteria were included.

A total of 269 patients diagnosed with PCP in Peking University First Hospital from April 2007 to October 2022 were retrieved through the electronic medical record system, excluding 5 HIV-positive patients, 22 patients under the age of 18, 34 patients with insufficient etiological evidence, 7 patients with incomplete clinical data, 1 patient with colonization, and 54 patients with non-RF. Finally, 146 patients meeting the criteria were included (**Figure 1**). In the overall population, the median age was 59 years (range 45.8-66 years), of which 98 (67.1%) were male and 48 (32.9%) were female. The median length of hospitalization was 22.5 days (range 12-34 days), and the total number of deaths during hospitalization was 57 (39%). Of the 83 patients admitted to ICU (56.8%), 44 (53%) died. In descending order, 58 cases (36.7%) suffered from autoimmune diseases, 42 cases (26.6%) from kidney diseases, 13 cases (8.2%) from organ transplantation and so on. There were 138 patients (94.5%) with a history of corticosteroid use and 84 patients (60.9%) with a history of corticosteroid withdrawal. The median corticosteroid use was 45 mg/d and the median duration of corticosteroid use was 71 days. There were 103 patients (70.5%) who had taken other immunosuppressive drugs.

The patients were divided into non-survivor (n=57, 39%) and survivor (n=89, 61%) according to death during hospitalization. Patients in the non-survivor group had a higher median age (62 vs 53, *P* < 0.001) and a lower percentage of patients with a history of chemotherapy (3.5% vs 14.6%, *P*=0.031). There were no statistically significant differences between the two groups in gender, underlying diseases, and treatment before onset of disease (**Table 1**).

**Table 1:** Demographics of non-*Human Immunodeficiency Virus* -*Pneumocystis* Pneumonia patients with respiratory failure patients.

	Total (n=146)	Non-survivor (n=57)	Survivor (n=89)	*P-*value
Age^a^, years	59 (45.8,66)	62 (53.5,71.5)	53 (38.5,63.5)	<0.001^*^
Sex, n (%) Male Female	98 (67.1) 48 (32.9)	35 (61.4) 22 (38.6)	63 (70.8) 26 (29.2)	0.239
Hospital admission, days	22.5 (12,34)	14 (8.5, 27.5)	25 (20, 39)	<0.001^*^
Underlying disease,n (%) Autoimmune disease Kidney disease Organ transplantation Solid malignant tumor Hematological diseases Skin disease Interstitial lung disease Primary immunodeficiency disease	58 (36.7) 42 (26.6) 13 (8.2) 12 (7.6) 12 (7.6) 16 (11) 4 (2.5) 1 (0.6)	27 (47.4) 17 (29.8) 2 (3.5) 5 (8.8) 2 (3.5) 5 (8.8) 1 (1.8) 0 (0)	31 (34.8) 25 (28.1) 11 (12.4) 7 (7.9) 10 (11.2) 11 (12.4) 3 (3.3) 1 (1.1)	0.131 0.821 0.067 1.000 0.177 0.498 0.949 1.000
Pre-admission immunosuppressants , n(%)				
Radiotherapy Chemotherapy Radiotherapy + chemotherapy	1 (0.7) 15 (10.3) 5 (3.4)	1 (1.8) 2 (3.5) 3 (5.3)	0 (0) 13 (14.6) 2 (2.2)	0.390 0.031^*^ 0.609
Corticosteroids Corticosteroids withdrawal Daily dosage at presentation ^a^ (mg) Corticosteroids duration ^a^ (days)	138 (94.5) 84 (60.9) 45 (35, 56.3) 71 (46.3,116)	55 (96.5) 33 (57.9) 45 (35,50) 68 (44,102)	83 (93.3) 51 (57.3) 45 (30,60) 72 (48,123)	0.642 0.944 0.784 0.425
In-hospital mortality, n (%)	57 (39)	/	/	/
ICU admission, n(%) ICU mortality	83 (56.8) 44 (53)	/ /	/ /	/ /

a Median (IQR); ^*^P<0.05, denotes statistically significant difference.

### Clinical course of *Pneumocystis* pneumonia with respiratory failure

In terms of clinical symptoms of non-HIV-PCP patients with RF, the proportions of dyspnea, fever, cough, sputum, chest pain and hemoptysis were not statistically different between the two groups, as shown in **Table 2**.

**Table 2:** Clinical features of non-*Human Immunodeficiency Virus* -*Pneumocystis* Pneumonia with respiratory failure patients.

	Non- survivor (n=57)	Survivor (n=89)	*P-*value
Symptom, n (%)			
Dyspnea	47 (82.5)	73 (82)	0.947
Fever	47 (82.5)	81 (91)	0.125
Cough	15 (26.3)	29 (32.6)	0.421
Expectoration	17 (29.8)	35 (39.3)	0.242
Chest pain	3 (5.2)	4 (4.5)	1.000
Hemoptysis	1 (1.8)	2 (2.2)	1.000
Laboratory findings			
Peripheral Blood			
White blood cell count ^a^ (×10^9/L)	8.64 (5.5, 12)	7.8 (4.9, 10)	0.200
Hemoglobin ^b^ (g/L)	104.6 ± 25.3	108.4 ± 23.5	0.357
Platelet count ^a^ (×10^9/L)	151 (93.5, 198)	171 (123.5, 241.5)	0.007^*^
Neutrophil count ^a^ (×10^9/L)	7.61(4.6, 10.6)	6.8 (4.2, 8.8)	0.123
Lymphocyte count ^a^ (×10^9/L)	0.4 (0.2, 0.9)	0.6 (0.4, 0.8)	0.069
NLR ^a^	17.3 (6.8, 30.6)	11.6 (7.1, 18.2)	0.018^*^
Albumin ^b^ (g/L)	27.5 ± 7.0	28.7 ± 5.1	0.227
Serum creatinine ^a^ (μmol/L)	100 (62.1, 157.5)	100.9 (70.7, 208.1)	0.334
Blood urea nitrogen ^a^ (mmol/L)	11 (5.9, 17.1)	8.8 (6.3, 15.8)	0.570
Lactate dehydrogenase ^a^ (IU/L)	486 (383.5, 826)	439 (317, 601)	0.022^*^
D-dimer ^a^ (mg/L)	1.0 (0.4, 2.6)	0.5 (0.3, 1.1)	0.013^*^
G-test ^a^	230 (6, 495.6)	139.1 (19.5, 350)	0.778
Procalcitonin (μg/L), n (%) <0.5 ≥0.5	35 (61.4) 19 (33.3)	59 (66.3) 22 (24.7)	0.321
Hypersensitive C-reactive protein ^a^ (mg/L)	80 (40.2, 117.3)	55 (18.2, 109.2)	0.076
Erythrocyte sedimentation rate ^b^ (mm/h)	54.8 ± 37.9	61 ± 32.2	0.336
CD3^+^ T cell count ^a^ (/μl)	301 (175, 574.7)	451.9 (259.4, 850.1)	0.011^*^
CD4^+^ T cell count ^a^ (/μl)	148.6(60.7, 225.6)	170.4 (103, 337.1)	0.094
CD8^+^ T cell count ^a^ (/μl)	156.4 (80.8, 280.6)	219 (129.2, 468)	0.005^*^
CD4/CD8 ^a^	0.9 (0.6, 1.6)	0.9 (0.5, 1.3)	0.333
Bronchoalveolar lavage fluid			
Macrophages ^a^(%)	28 (15, 49)	36 (18.5, 49.5)	0.230
Neutrophil ^a^ (%)	55 (28, 74)	29 (11, 52.5)	0.001^*^
Lymphocyte ^a^ (%)	16 (7, 23)	25 (12, 47)	0.001^*^

^a^Median (IQR); *P<0.05, denotes statistically significant difference. NLR, Neutrophil/Lymphocyte Ratio.

**Figure 2:**
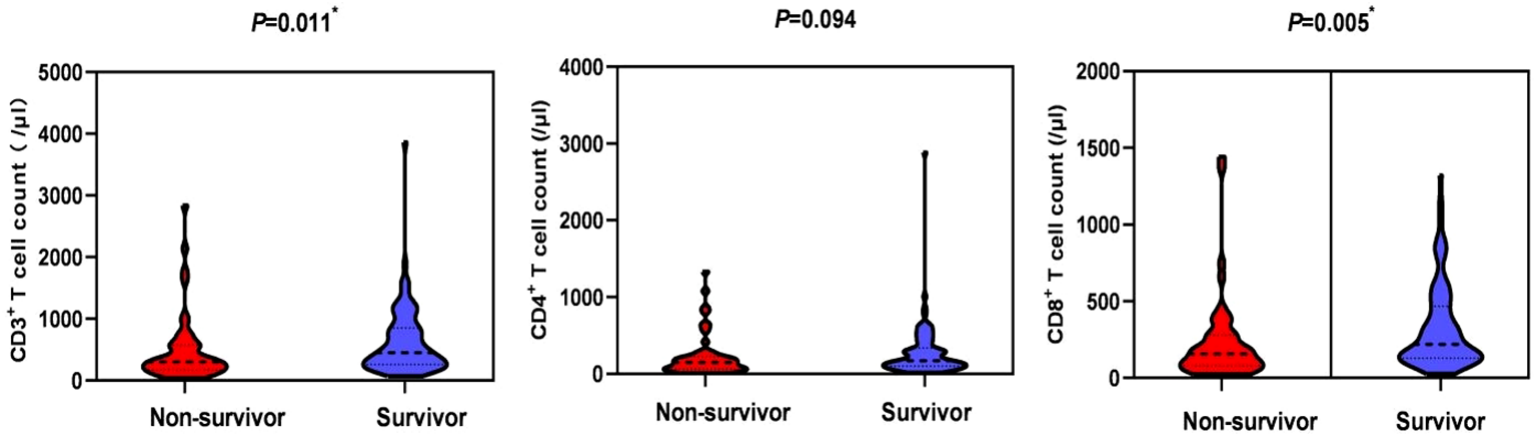
Comparison of peripheral blood lymphocyte subsets in the non-survival group and the survival group. *P < 0.05, indicating statistically significant difference.

**Figure 3:**
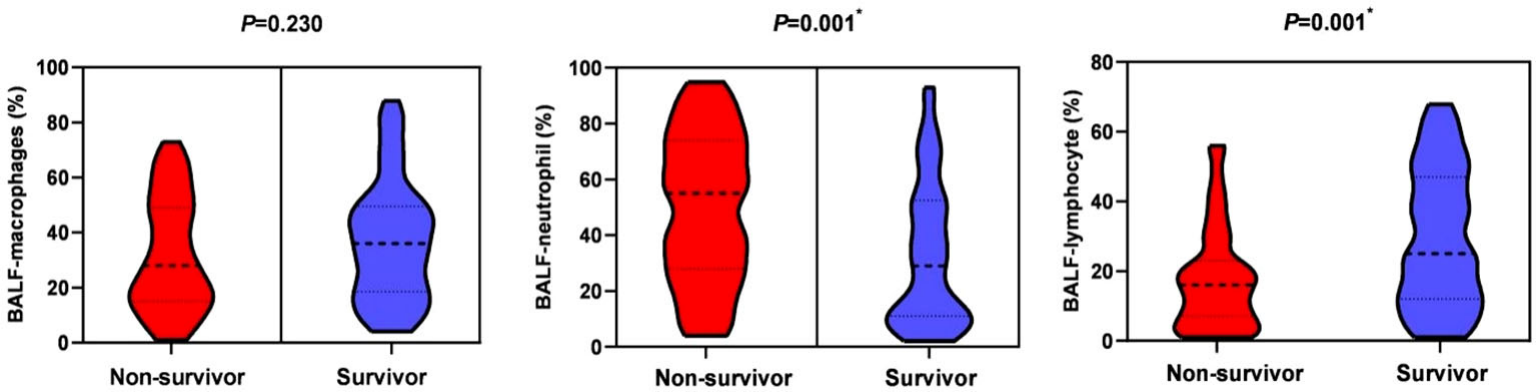
Bronchoalveolar lavage fluid-cytological classification in the non-survival group and the survival group. *P < 0.05, indicating statistically significant difference.

In terms of laboratory measures at admission, patients in the non-survivor group had lower platelet counts (151 vs 171, *P*=0.007), neutrophil-lymphocyte ratio (NLR) (17.3 vs 11.6, *P*=0.018), lactate dehydrogenase (LDH) (486 vs 439, *P*=0.022), and D-dimer (1.0 vs 0.5, *P*=0.013) was higher, and there was no statistical difference in inflammatory indexes between the two groups. In terms of immunological indicators, peripheral blood CD3^+^ T cell count (301 vs 451.9, *P*=0.011) and CD8^+^ T cell count (156.4 vs 219, *P*=0.005) were lower in the non-survivors group than in the survivor group, as shown in **Figure 2**. In BALF, patients in the non-survivor group had a higher percentage of neutrophils (55% vs 29%, *P*=0.001) and a lower percentage of lymphocytes (16% vs 25%, *P*=0.001), as shown in **Figure 3**.

Etiological examination results showed that patients in the non-survivor group had a higher proportion of co-infection with pathogens than those in the survivor group (82.5% vs 58.4%, *P*=0.002) and a higher proportion of co-infection with two or more pathogens (47.4% vs 19.1%, *P*<0.001) and a higher proportion of hospital-acquired pneumonia-associated pathogens (42.1% vs. 11.2%, *P*<0.001). There was no significant difference in the proportion of *Cytomegalovirus* (CMV) infection and *Aspergillus* infection between the two groups.

In terms of imaging, patients in the death group had a higher proportion of pneumothorax than those in the survivor group (17.5% vs 4.5%, *P*=0.020), while there was no statistical difference between the two groups in the proportion of ground glass density shadows, solid shadows, mesh shadows, cellular shadows, and mediastinal emphysema, as shown in **Table 3**.

**Table 3:** Clinical features of non-*Human Immunodeficiency Virus* -*Pneumocystis* Pneumonia with respiratory failure patients.

	Non-survivor (n=57)	Survivor (n=89)	*P-*value
Pathogen infection			
Combined with one type of pathogen infection Bacteria Fungi Virus Infection with ≥ two pathogens Bacteria + fungi Fungi + virus Bacteria + virus Bacteria + fungi + virus	47(82.5) 20(35.1) 5(8.8) 5(8.8) 10(17.5) 27(47.4) 9(15.8) 3(5.3) 8(14) 7(12.3)	52(58.4) 35(39.3) 6(6.7) 5(5.6) 24(27) 17(19.1) 3(3.3) 5(5.6) 8(9) 1(1.1)	0.002^*^ 0.606 <0.001^*^
Type of pathogen infection, n (%) * Cytomegalovirus* * Aspergillus* Pathogens associated with hospital acquired pneumonia	25(43.9) 12(21.1) 24(42.1)	35(39.3) 13(14.6) 10(11.2)	0.587 0.313 <0.001^*^
Imaging features			
Ground-glass opacity, n (%)	46(80.7)	80(89.9)	0.115
Consolidation, n (%)	31(54.4)	51(57.3)	0.729
Grid shadow, n (%)	5(8.8)	5(5.6)	0.462
Honeycomb, n (%)	7(12.3)	5(5.6)	0.153
Pneumothorax, n (%)	10(17.5)	4(4.5)	0.009^*^
Mediastinal emphysema, n (%)	6(10.5)	2(2.2)	0.076

a : median (quartile), ^b^: mean ± standard deviation; ^*^P < 0.05, indicating statistically significant difference.

In terms of treatment, the proportion of second-line treatment in the non-survivor group was significantly higher than that in the survivor group (36.8% vs 11.2%, *P* < 0.001), and the proportion of Caspofungin (56.1% vs 38.2%, *P*=0.034) and the proportion of high-dose corticosteroids (≥1mg/(kg·d)) (56.6% vs 31.3%, *P*=0.003) were higher than those in the survivor group, as shown in **Supplementary Figure S1**. The rates of high-flow oxygen therapy, non-invasive mechanical ventilation, invasive mechanical ventilation and ICU admission in the death group were significantly higher than those in the survivor group (*P*<0.001), but the length of ICU stay was not statistically different between the two groups, as shown in **Table 4**.

**Table 4:** Treatments of non-*Human Immunodeficiency Virus* -*Pneumocystis* Pneumonia with respiratory failure patients.

	Non-survivor (n=57)	Survivor (n=89)	*P-*value
Treatments			
TMP-SMZ (mg/(kg·d))			0.566
≥15, n (%) <15, n (%)	46(85.2) 8(14.8)	77(88.5) 10(11.5)	
Second-line treatment, n (%)	21(36.8)	10(11.2)	<0.001^*^
Caspofungin, n (%)	32(56.1)	34(38.2)	0.034^*^
Corticosteroid therapy (mg/(kg·d))			0.003^*^
<1, n (%)	23(43.4)	57(68.7)	
≥1, n (%)	30(56.6)	26(31.3)	
Oxygen therapy, n (%)			
High flow oxygen therapy	46(80.7)	51(57.3)	0.003^*^
Non-invasive mechanical ventilation	48(84.2)	28(31.5)	<0.001^*^
Invasive mechanical ventilation	47(82.5)	9(10.1)	<0.001^*^
ICU admission, n (%)	44(77.2)	39(43.8)	<0.001^*^
Length of stay in ICU ^a^ (days)	11(5.3,17.8)	14(6,22)	0.393

a : median (IQR); ^*^P < 0.05, indicating statistically significant difference.

### Important events of the no-survivor group and survivor group during hospitalization

**Figure 4:**
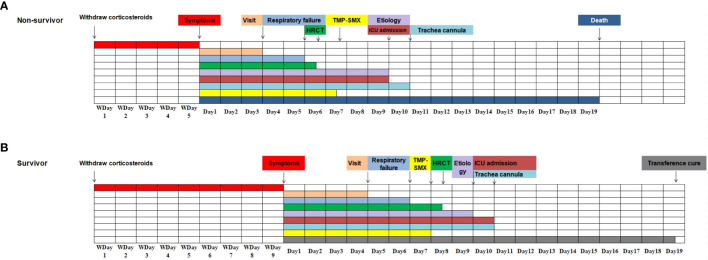
Progression and associated event notes in non-*Human Immunodeficiency Virus -Pneumocystis *Pneumonia patients with respiratory failure (all event notes are represented by median). **(A)**. The progression of the disease and related important events in the non-survivor group; **(B)**. The progression of the disease and related important events in the survivor group.

Compared with the survival group, a higher proportion of patients in the death group had less than 5 days of withdrawal from corticosteroids to onset of symptoms (57.6% vs 25.5%, *P*=0.003), and the time between treatment and initiation of sulfonamides therapy was longer (3 vs 2, *P*=0.019), as shown in **Table 5** and **Figure 4**. The effect of prolonged treatment on in-hospital mortality was observed according to the interquartile segment of sulfonamides treatment interval, and the results showed that in-hospital death of non-HIV-PCP patients with RF increased with prolonged medication duration (*P*=0.021).

**Table 5:** Comparison of critical event nodes in non-*Human Immunodeficiency Virus* -*Pneumocystis* Pneumonia with respiratory failure patients.

	Non-survivor (n=57)	Survivor (n=89)	*P-*value
Time from withdrawal of corticosteroids to onset of symptoms ^a^ (days)	5 (2, 12)	9 (5, 13)	0.102
≤5, n (%) >5, n (%)	19 (57.6) 14 (42.4)	13 (25.5) 38 (74.5)	0.003^*^
Time from illness onset to the visit ^a^ (days) ≤4, n (%) >4, n (%)	3 (0, 7) 36 (63.2) 21 (36.8)	4 (0, 10) 46 (51.7) 43 (48.3)	0.124 0.173
Time from illness onset to respiratory failure^a^ (days) ≤14, n (%) >14, n (%)	5 (2, 8.5) 53 (93) 4 (7)	6 (3, 12) 73 (82) 16 (18)	0.219 0.060
Time from illness onset to HRCT ^a^ (days)	5.5 (1.8, 11)	7.5 (4, 13)	0.062
≤4, n (%) >4, n (%)	23 (46) 27 (54)	26 (29.5) 62 (70.5)	0.052
Time from illness onset to diagnosis ^a^ (days)	9 (5, 15)	9 (6, 16)	0.448
≤10, n (%) >10, n (%)	31 (54.4) 26 (45.6)	50 (56.2) 39 (43.8)	0.832
Time from illness onset to ICU ^a^ (days)	9 (4, 14)	10 (4, 17)	0.661
≤9, n (%) >9, n (%)	26 (59) 18 (41)	19 (48.7) 20 (51.3)	0.344
Time from illness onset to trachea cannula ^a^ (days)	10 (7, 17)	10 (5, 17.5)	0.947
≤14, n (%) >14, n (%)	31 (66) 16 (34)	7 (77.8) 2 (22.2)	0.760
Time from illness onset to treatment with TMP-SMX ^a^ (days)	6.5 (4, 11)	7 (4.5, 14)	0.199
≤4, n (%) >4, n (%)	21 (37.5) 35 (62.5)	22 (24.7) 67 (75.3)	0.101
Time from visit to initiation of TMP-SMX therapy ^a^ (days) <2, n (%) ≥2, n (%)	3 (2, 5) 10 (17.9) 46 (82.1)	2 (1, 4) 38 (42.7) 51 (57.3)	0.019^*^ 0.002^*^
Time from illness onset to death ^a^ (days)	19 (15.5, 30.5)	/	/
Time from illness onset to symptoms disappear ^a^ (days)	/	18.5 (13, 27)	/

a : median (IQR); *P < 0.05, indicating statistically significant difference.

### Univariate and multivariate analysis of in-hospital death in non-HIV-PCP patients with RF

The optimal thresholds for CD4^+^ T cell count and CD8^+^ T cell count were determined by ROC curve to be 232/μl and 115/μl, respectively, as shown in **Supplementary Figure S2**. In univariable analysis, age ≥60 years old, chemotherapy history, platelet count <100×10^9/L, NLR≥20, LDH≥454 IU/L, CD4^+^ T cell count < 232/μl, CD8^+^ T cell count <115/μl, percentage of BALF-neutrophils ≥50%, percentage of BALF-lymphocytes <20%, infection with hospital-acquired pneumonia-associated pathogens, pneumothorax, withdrawal of corticosteroids to the onset of symptoms ≤5 days, and time from visit to initiation of sulfonamides therapy ≥2 days were associated with prognosis in non-HIV-PCP patients with RF (*P* < 0.05).

**Figure 5:**
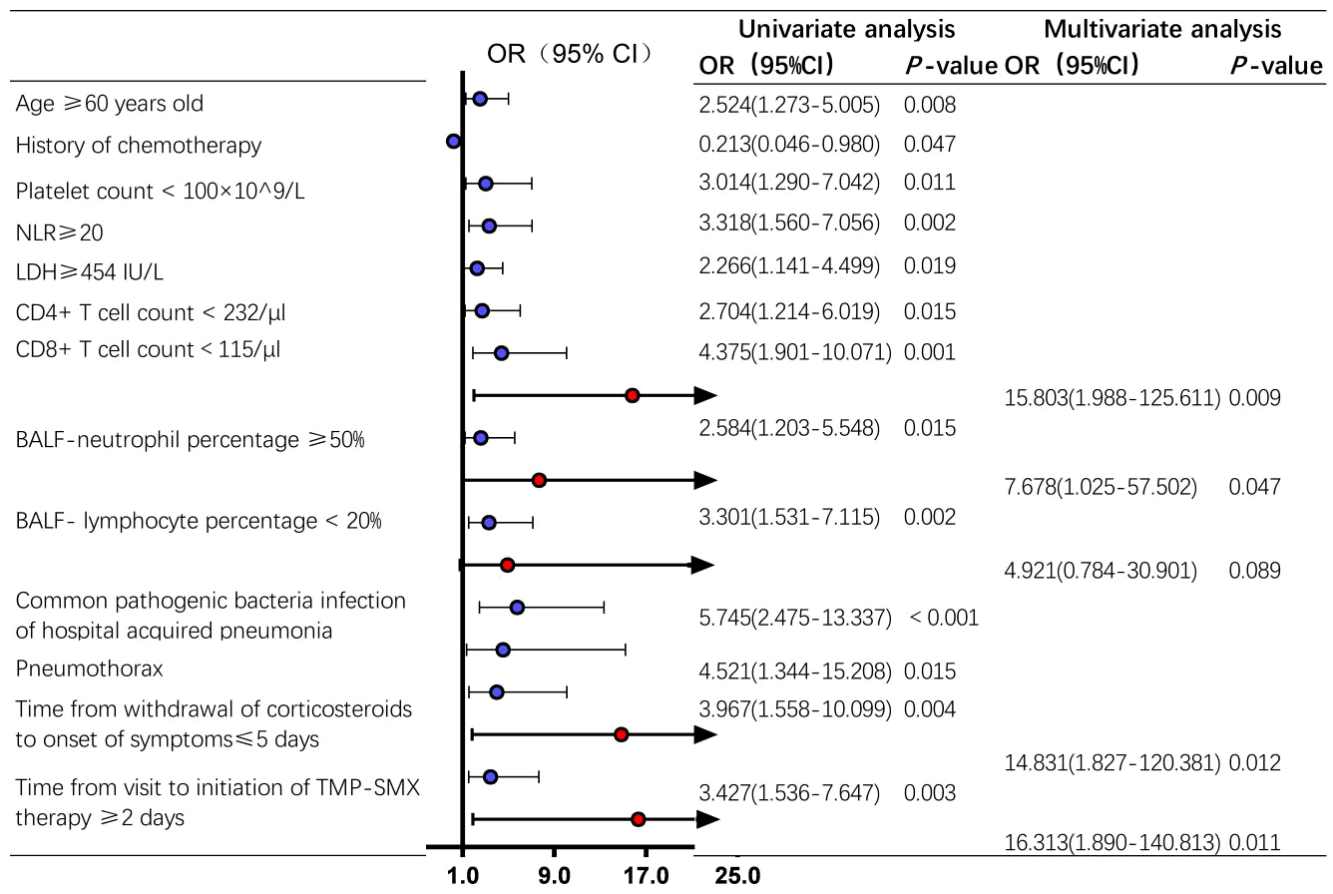
Analysis of risk factors for death during hospitalization in non-*Human Immunodeficiency Virus* -*Pneumocystis* Pneumonia patients with respiratory failure - Forest map. Blue represents the single factor analysis index, red represents the multi-factor analysis index; CI, Confidence Interval; OR, Odds Ratio.

Factors with *P <*0.1 were incorporated into the logistic model for multivariate analysis, and the results showed that CD8^+^ T cell count <115/μl (OR=15.803, 95%CI 1.988-125.611, *P*=0.009), BALF-neutrophils percentage ≥50%(OR=7.678, 95%CI 1.025-57.502, *P*=0.047), withdrawal of corticosteroids to the onset of symptoms ≤5 days (OR=14.831, 95%CI 1.827-120.381, *P*=0.012), and the time from presentation to initiation of sulfonamides ≥2 days (OR=16.313, 95%CI 1.890-140.813, *P*=0.011) was an independent risk factor for in-hospital death in PCP patients with RF, as shown in **Figure 5**.

### Univariate and multivariate analysis of 90-day prognosis in non-HIV-PCP patients with RF

In this study, 146 non-HIV-PCP patients with RF were followed up for 90 days, 9 were lost to follow-up and 58 were died (57 of them died during hospitalization and 1 died during follow-up). A total of 137 patients completed follow-up, and the 90-day survival rate was 57.7%. The survival analysis curve is shown in **Supplementary Figure S3**. Univariable analysis was performed for T cell count, BALF-neutrophil percentage, BALF-lymphocyte percentage, hospital-acquired pneumonia-associated pathogen infection, pneumothorax, time from withdrawal of corticosteroids to onset of symptoms, and time from visit to initiation of sulfonamides use.

**Figure 6:**
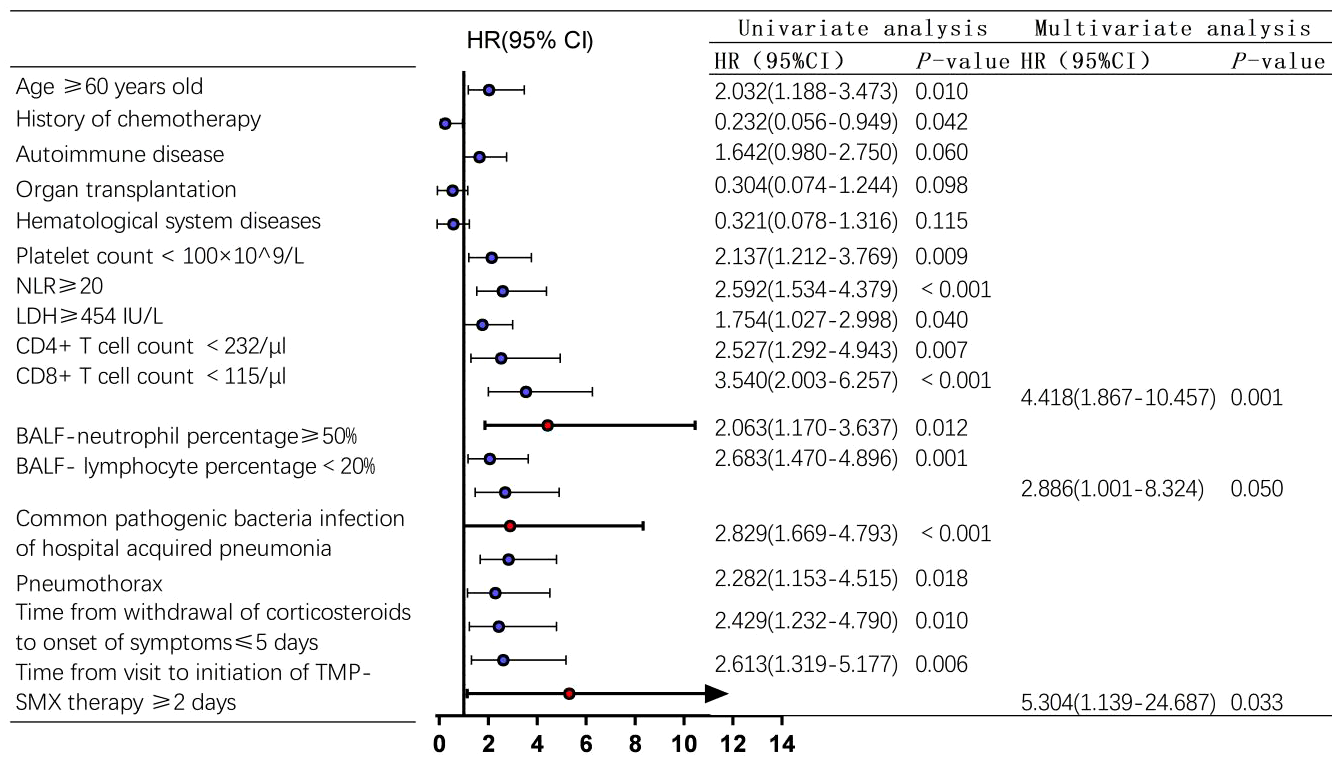
Analysis of 90-day risk factors for all-cause death in non-*Human Immunodeficiency Virus* -*Pneumocystis* Pneumonia patients with respiratory failure-Forest map. blue represents the single factor analysis index, red represents the multi-factor analysis index; CI, Confidence Interval; HR, Hazard Ratio.

Factors with *P* < 0.1 were included in cox regression model for multivariate analysis, and the results showed that a CD8^+^ T cell count <115/μl (HR=4.418, 95%CI 1.867-10.457, *P*=0.001) and the time from visit to initiation of sulfonamides therapy ≥2 days (HR=5.304, 95%CI 1.139-24.687, *P*=0.033) were independent risk factors for 90-day all-cause death in non-HIV-PCP patients with RF, as shown in **Figure 6**.

## Discussion

In non-HIV patients, PCP-related RF remains a highly lethal disease, with a mortality rate of 50-80% (Festic et al., 2005; Limper et al., 2017). In this study, the mortality rate of non-HIV-PCP patients with RF was 39%, which was lower than that reported in previous literature. The main reason was considered that the enrolled population in the previous study was mostly RF patients who required mechanical ventilation and were admitted to ICU (Festic et al., 2005; Boonsarngsuk et al., 2009; Ko et al., 2014). Studies have shown that patients requiring mechanical ventilation have a higher mortality rate (Festic et al., 2005; Ko et al., 2014), which increases the overall mortality rate. By analyzing the clinical course of non-HIV-PCP patients with RF, this study innovatively explored the relationship between important event nodes and prognosis in PCP patients with RF, and screened out the risk factors affecting prognosis.

T lymphocyte subsets are important prognostic markers for non-HIV-PCP patients with RF. Previously, most of the studies were related to T lymphocyte subsets and PCP, and the focus was on CD4^+^ T cells. Previous studies have shown that approximately 90% of PCP occurs in patients with a CD4^+^ T cell count <200/μl (Helweg-Larsen et al., 1998), and the HIV-related guidelines have clearly recommended prophylactic treatment of PCP with a CD4^+^ T cell count < 200/μl as a level IA recommendation. However, the role of CD4^+^ T cells in non-HIV-PCP patients is not as prominent, and they are mainly valuable for identifying specific individuals at particularly high clinical risk of PCP, such as recipients of allogeneic hematopoietic stem cell (Castagnola et al., 1995), patients with solid malignancies undergoing chemotherapy (Sepkowitz et al., 1992), organ transplantation patients, and individuals with hematological malignancies (Mansharamani et al., 2000a). Tang et al. conducted a study on immunological indicators for immunocompromised patients and found that the decrease in the number of CD3^+^ T, CD4^+^ T and CD8^+^ T cells was the most typical feature of PCP patients (Tang et al., 2021). Brakemeier and colleagues observed similar results in laboratory measurements of PCP patients who received organ transplants, supporting CD3^+^ T and CD8^+^ T cell counts as prominent predictors of the development of non-HIV-PCP (Brakemeier et al., 2018). Furthermore, in patients with autoimmune diseases, a CD3^+^ T cell count <625/μl has been identified as an independent predictor of PCP, while a CD8^+^ T cell count <160/μl is a risk factor for death in PCP patients (Li et al., 2017). In our study, the majority of the subjects were patients with inflammatory diseases. The results showed that CD4^+^ T cell count was associated with the prognosis of non-HIV-PCP patients with RF, but was not an independent risk factor. On the other hand, a CD8^+^ T cell count < 115/μl was identified as an independent risk factor for all-cause death during hospitalization and 90 days in non-HIV-PCP patients with RF. This highlights the important role of CD8^+^ T cells in the host immune response. Previous studies have suggested that the pathogenesis of CD8^+^ T cells and non-HIV-PCP is as follows:

In the absence of CD4^+^ T cells, CD8^+^ T cells will play a role in eliminating *Pneumocystis* and constitute a secondary defense against the fungus. The protective factor reacts to *Pneumocystis* in absence of CD4^+^ T cells (Beck et al., 1996; Ruan et al., 2016). In cases of PCP, a large number of CD8^+^ T cells are recruited to the lungs, especially when CD4^+^ T cell depletion is significant (Meissner et al., 2005). While the removal of CD8^+^ T cells did not affect fungal load in immunocompetent mice, removing of CD8^+^ T cells in the absence of CD4^+^ T cells led to an increase in *Pneumocystis* load (Beck et al., 1996; Wright et al., 1999). Conversely, the increasing the number of CD8^+^ T cells through IL-7 in mice with depleted CD4^+^ T cells further reduced fungal load (Ruan et al., 2016). Studies have shown that CD8^+^ T cells eliminate *Pneumocystis* through cytokines such as TNF-α, TNF-β or IFN-γ (Mosmann and Sad, 1996; Mosmann et al., 1997), as well as cytotoxic efflux molecules like perforin or granase. CD8^+^ T cells also exhibit a pattern similar to helper T cell (Th) 1 and Th2, called cytotoxic T cells (Tc) 1 and Tc2. McAllister et al. discovered that in hosts with deficient CD4^+^ T cells, Tc1 is crucial for the clearance of *Pneumocystis*. In contrast, CD8^+^ T lymphocytes without the Tc1 phenotype (Tc2) seem to cause tissue damage (McAllister et al., 2004). The mechanism is complex, and further research on CD8^+^ T cell subsets is necessary to determine the specific effects of different subsets of CD8^+^ T cells on PCP. Our results also indicate a correlation between CD4^+^ T and CD8^+^ T cell levels and PCP outcomes. This serves as a reminder that patients with chronic inflammatory or autoimmune diseases should not only focus on monitoring CD4^+^ T cell count but also pay attention to CD8^+^ T cell count. This may have significant implications for disease prevention, treatment, and prognosis assessment.

Several studies have indicated that the percentage of neutrophils in BALF can serve as a prognostic marker for non-HIV-PCP patients (Smith et al., 1988; Mason et al., 1989). Lee et al. discovered that a 10% increase in BALF-neutrophil levels correlated with a 15% and 21% increase in 30-day and 60-day mortality, respectively (Lee et al., 2015). Tamai et al. found that BALF-neutrophils ≥31% were significantly associated with in-hospital mortality in non-HIV-PCP patients (Tamai et al., 2014). In our study, we observed that BALF-neutrophils ≥50% were associated with in-hospital death in non-HIV-PCP patients with RF, which is similar to previous findings suggesting that neutrophils may be associated with lung injury, RF and death (Thomas and Limper, 2004). Furthermore, our study demonstrated that BALF-lymphocytes were linked to 90-day all-cause death during hospitalization for non-HIV-PCP patients with RF in the univariate analysis. Some studies have only explored the relationship between BALF-lymphocytes and the prognosis of PCP. Kim et al. reported that BALF-lymphocyte ≤45% is associated with failure of first-line therapy using TMP-SMX (Kim et al., 2018). Additionally, Chung et al. demonstrated a significant correlation between BALF-lymphocyte percentage ≤30% and 90-day all-cause mortality in PCP patients (Chung et al., 2022). Hirasawa et al. reported that BALF-lymphocyte percentage ≥20% was significantly associated with increased 90-day and 1-year survival in patients with acute respiratory failure (ARF) caused by interstitial lung disease or non-interstitial lung disease (Hirasawa et al., 2021). These findings suggested that BALF-lymphocytes may play a vital role in the prognosis of PCP patients with RF, and the usefulness of BALF-lymphocytes warrants further investigation.

In addition to, it is also crucial to pay attention to important clinical events during the diagnosis and treatment of the disease, as well as identify any gaps in the diagnostic process. Previous studies have demonstrated that non-HIV patients experience a longer time interval between admission and initiation of PCP treatment compared to HIV-infected patients. The delay in treatment may be attributed to the non-specific clinical manifestations of PCP, leading to a delay in diagnosis (Li et al., 2014). Roux and colleagues also reported that non-HIV-PCP patients had a longer time between admission to initial TMP-SMX treatment and a higher 90-day mortality rate compared to HIV-PCP patients (Roux et al., 2014). KO et al. found that age and initial anti-PCP treatment failure were independently associated with increased mortality (Ko et al., 2019). Song et al. found that in patients with rheumatic disease and PCP, initiating treatment with TMP-SMX 7 days after the onset of symptoms was significantly linked to a higher 90-day mortality (Song et al., 2022). These findings highlight the fact that delayed diagnosis and treatment are major contributors to increased mortality and the need for mechanical ventilation in PCP patients. Additionally, early empiric anti-PCP treatment remains a topic of debate. We found that the median difference in the time to start TMP-SMX treatment between non-survivors and survivors was only 1 day, but this difference was significantly associated with in-hospital death and 90-day death in non-HIV-PCP patients with RF, and anti-PCP treatment within 2 days of admission was associated with improved outcomes. This finding is of significant importance for clinicians, emphasizing the need for immediate treatment of suspected PCP, rather than waiting for a confirmed diagnosis.

In the cohort of non-HIV-PCP patients with RF, 94.5% of the patients received corticosteroids within the previous 1 month, and 60.9% of the patients developed symptoms during corticosteroids withdrawal. We innovatively found that the time from corticosteroids withdrawal to symptom onset was correlated with disease prognosis, and when the time from corticosteroids withdrawal to symptom onset was less than 5 days, the prognosis was worse than that of patients > 5 days. In the process of managing clinical patients, most of the focus is on the dosage and duration of corticosteroids use, with little attention paid to corticosteroids withdrawal. The importance of prophylactic use of TMP-SMX for 4 weeks or more with a prednisone equivalent dose of corticosteroids ≥20mg/day and other conditions leading to immunosuppression is now recognized (Sepkowitz, 2002; Thomas and Limper, 2004; Huang et al., 2006; Limper et al., 2011). However, in addition to monitoring the dosage and time of corticosteroids use, attention should also be given to the situation of corticosteroids withdrawal. Our study demonstrated that the faster the disease onset after corticosteroids withdrawal, the worse the prognosis for patients. This finding may indirectly reflect the host immunity of PCP patients and assist doctors in understanding the progression of the disease and choosing more effective treatment options. Currently, the occurrence and development patterns of past corticosteroids use, corticosteroids withdrawal in non-HIV-PCP patients are not clear. This study innovatively explored the effects of clinically important event nodes on in-hospital death and 90-day prognosis of non-HIV-PCP patients with RF.

However, this study has some limitations. Firstly, due to its the retrospective study, not all patients were tested for relevant laboratory indicators, such as inflammatory markers and T lymphocyte subsets. Therefore, the role of these indicators in predicting in-hospital death and 90-day death may have been underestimated. Secondly, late presentation to the hospital and poor patient compliance may have contributed to poor clinical outcomes for some patients. Therefore, in the future, we will conduct prospective studies to further explore the factors that influence patient prognosis.

## Conclusions

In conclusion, several factors indicate a poor prognosis in non-HIV-PCP patients with RF. These factors include a low CD8^+^ T cell count in peripheral blood, a high percentage of BALF-neutrophils, a short time from corticosteroids withdrawal to symptom onset, and a long time from visit to initiation of TMP-SMX therapy.

## Data availability statement

The original contributions presented in the study are included in the article/**Supplementary Material**. Further inquiries can be directed to the corresponding authors.

## Ethics statement

The study involving human participants was reviewed and approved by the Ethics Committee of Peking University First Hospital.

## Author contributions

JL: Data curation, Formal analysis, Writing – original draft. XM: Supervision, Writing – review & editing. HL: Conceptualization, Supervision, Validation, Visualization, Writing – review & editing. XL: Project administration, Resources, Supervision, Writing – review & editing.
