# Supplementary material for: Clinical course and prognostic factors of Pneumocystis pneumonia with respiratory failure in non-HIV patients
Source: Front Cell Infect Microbiol. 2024 Jul 10;14:1380494. doi: 10.3389/fcimb.2024.1380494 (PMC11270599; doi:10.3389/fcimb.2024.1380494)
Supplement: Supplementary file 1 [file DataSheet_1.docx]

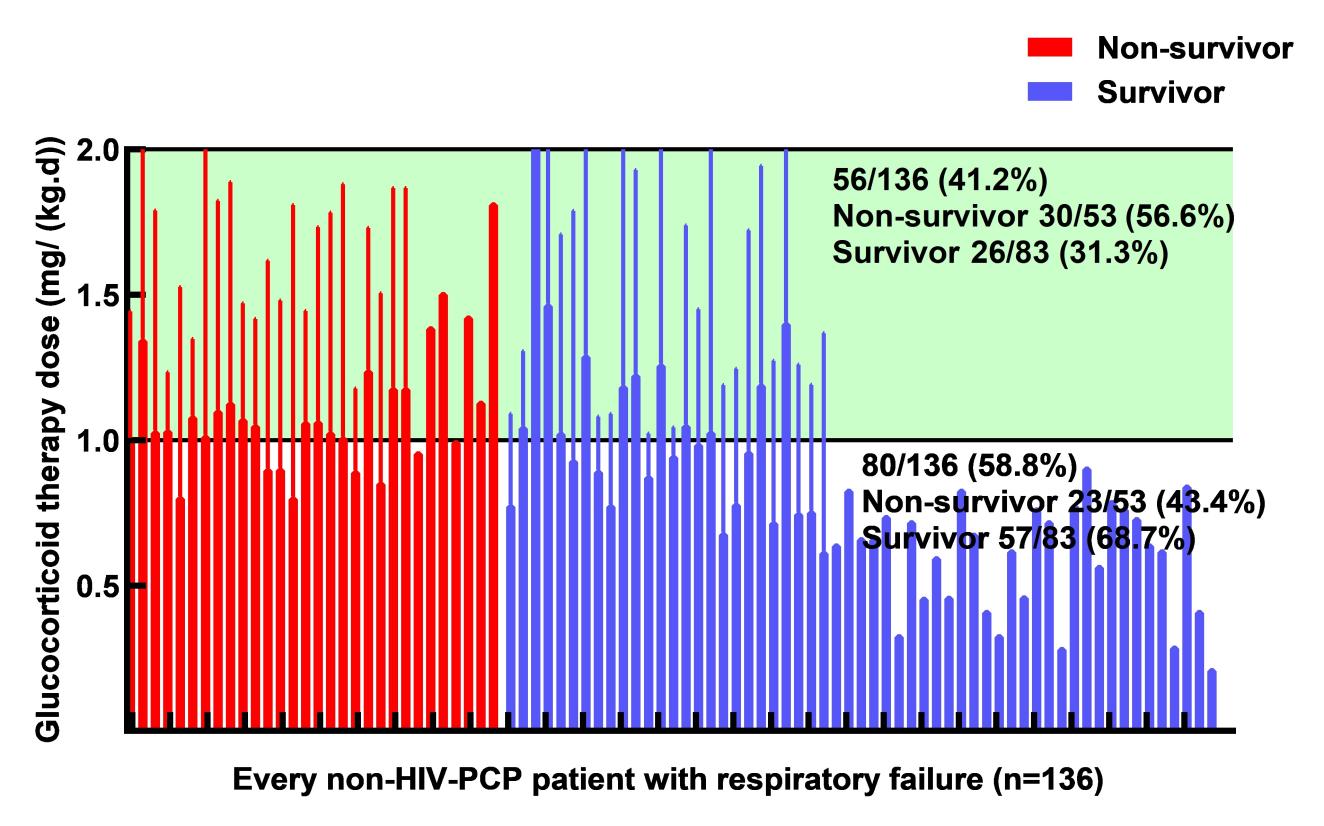


**Figure S1.** **Individual dose bars of corticosteroid therapy in 136 non-HIV-PCP patients**

#### **Note:** The green area represented corticosteroid dose ≥1 mg/ (kg·d) (41.2%), including 30 patients (56.6%) in the non-survivor group and 26 patients (31.3%) in the survivor group. The white area indicated a dose of corticosteroid <1 mg/ (kg·d) (58.8%), including 23 patients in the non-survivor group (43.4%) and those in the survivor group (68.7%).


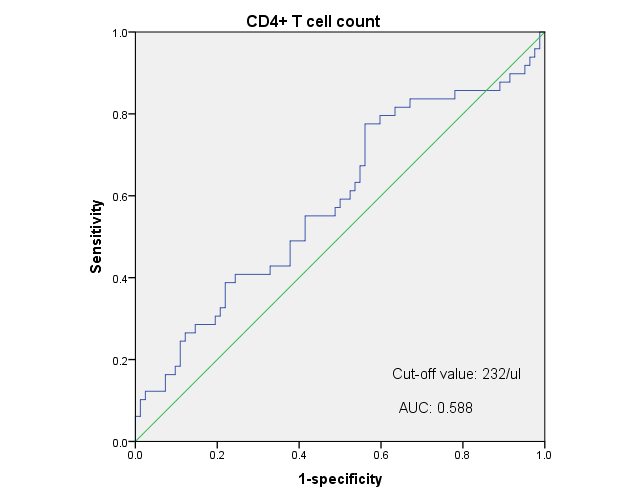

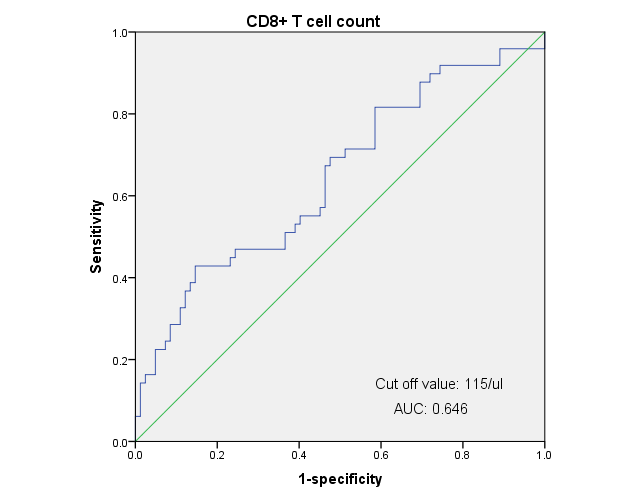


**(A) (B)**

**Figure S2. The optimal threshold is determined by the ROC curve.**

**Note: (A)** CD4+ T cell count, **(B)** CD8+ T cell count


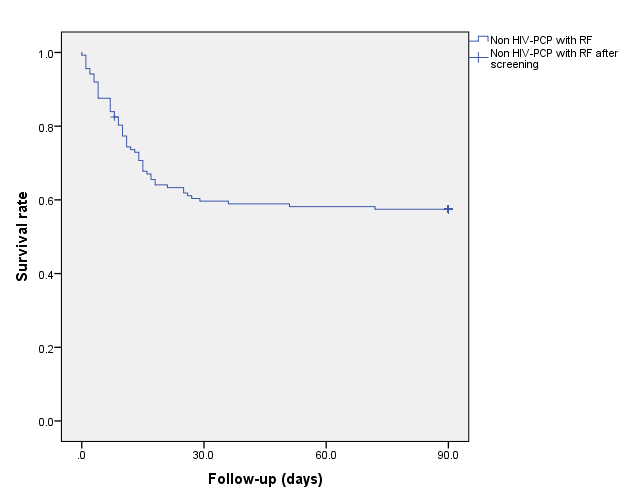


**Figure S3. Overall survival curve of non-HIV-PCP patients with RF (Kaplan-Meier curve)**
